# Meta-evaluation of a participatory process in the strengthening of municipal management

**DOI:** 10.11606/S1518-8787.2017051007047

**Published:** 2017-11-13

**Authors:** Cristiane Andrea Locatelli de Almeida, Oswaldo Yoshimi Tanaka

**Affiliations:** I Universidade de São Paulo. Faculdade de Saúde Pública. Programa de Pós-Graduação em Saúde Pública. São Paulo, SP, Brasil; II Universidade de São Paulo. Faculdade de Saúde Pública. Departamento de Política, Gestão e Saúde. São Paulo, SP, Brasil

**Keywords:** Health Evaluation, methods, Process Assessment (Health Care, Participative Planning, Health Manager, Decision Making, Health Services Administration, Qualitative Research, Avaliação em Saúde, métodos, Avaliação de Processos (Cuidados de Saúde, Planejamento Participativo, Gestor de Saúde, Tomada de Decisões, Administração de Serviços de Saúde, Pesquisa Qualitativa

## Abstract

**OBJECTIVE:**

To evaluate, with a focus on participation, an evaluation process developed by municipal managers and administrators of a health region in the state of São Paulo, considering the need for theoretical reflection on participatory health practices in the Brazilian context.

**METHODS:**

Qualitative research that used the framework developed by Daigneault and Jacob (2009) to analyze the empirical material, encompassing three dimensions of participation: control of the evaluation process, diversity of participants, and extent of their involvement. We highlighted decisions or contextual aspects that deepened or limited the participatory option in the process under study.

**RESULTS:**

We identified the presence and important performance of stakeholders who are “not specialists in evaluation”, through participation both in the direction of the evaluation process and in its distinct stages. The formed group started from their own annoyances added to the need for information and reflection to define the subject and scope of the evaluation; the use of the process planned by them guided the definition of the data to be collected and the format of result dissemination; the empirical material analysis was undertaken jointly by the participants. Regarding the third dimension, a limitation was identified regarding the diversity of actors involved due to the prioritization of the possibility of in-depth work with a fixed group of managers.

**CONCLUSIONS:**

It is stated that there is no “ideal participation model” for evaluations. In certain contexts and structures, real opportunities for participation – even if they seem fragile at first sight – should be leveraged, and that requires flexibility and critical reflection on the part of those responsible for the evaluation processes to undertake the necessary adjustments.

## RESUMO

**OBJETIVO:**

Meta-avaliar, com foco na participação, um processo avaliativo desenvolvido por secretários e assessores municipais de uma região de saúde do estado de São Paulo, tendo em vista a necessidade de reflexão teórica sobre práticas de avaliação participativa na área da Saúde no contexto brasileiro.

**MÉTODOS:**

Pesquisa qualitativa que utilizou, para análise do material empírico, o referencial desenvolvido por Daigneault e Jacob (2009), englobando três dimensões da participação: direção do processo avaliativo, diversidade dos participantes e extensão do envolvimento dos mesmos. Foram destacadas decisões ou aspectos contextuais que fizeram com que a opção participativa fosse aprofundada ou limitada no processo em foco.

**RESULTADOS:**

Identificou-se a presença e importante atuação de atores interessados “não especialistas em avaliação”, por meio da participação tanto na direção do processo avaliativo como em suas diferentes etapas. O grupo formado partiu de seus próprios incômodos somados à necessidade de informação e reflexão para a definição do tema e escopo da avaliação; a utilização do processo por eles planejada guiou a definição dos dados a serem coletados e o formato da disseminação dos resultados; a análise do material empírico foi empreendida de forma conjunta pelos participantes. No tocante à terceira dimensão, foi identificada uma limitação quanto à diversidade de atores envolvidos em função da priorização da possibilidade de trabalho aprofundado com um grupo fixo de gestores.

**CONCLUSÕES:**

Afirma-se que não existe um “modelo ideal de participação” para avaliações. Em determinados contextos e estruturas, oportunidades reais de participação – mesmo que pareçam frágeis à primeira vista – devem ser aproveitadas, exigindo, para tanto, flexibilidade e reflexão crítica por parte dos responsáveis pelos processos avaliativos, visando a empreender as adequações que se fizerem necessárias.

## INTRODUCTION

The issue related to the quality of programs and policies evaluation carried out has encouraged evaluators in Brazil to reflect on their practices^13^ ^,^ ^14^ ^,^ ^18^. Societies and associations in several countries have defined and published guides and standards^2^ ^,^ ^24^ ^,^ ^26^ aimed at guiding meta-evaluations – processes in which the quality of evaluations is evaluated^20^ ^,^ ^23^ – and contribute to its improvement.

These documents are quite general and comprehensive^5^, given the need to point out ethical and technical rigor principles to a wide variety of practices that make up the contemporary scenario of the area. The question arises as to how to bring more specific parameters to evaluate concrete initiatives.

Some authors, such as Furtado et al.^15^, suggest that criteria related to the characteristics of the evaluations developed in their contexts be added to the international references, to allow for reflection and judgment on their quality.

In this study, adopting such proposal, we highlight the criterion “participation” for the meta-evaluation, since the basis of the discussion is the use of the participatory methodology in the evaluation of public programs.

In Brazil, participative approaches for evaluation in the health sector are starting to gain space in the literature, referenced by experiences with the inclusion of stakeholder groups in the evaluation processes^1^ ^,^ ^6^, with the insertion of the participatory evaluation in the fabric of wider social intervention processes^3^, and with considering the criterion “participation” in meta-evaluations^14^. We deem it fundamental to continue this journey in the search for an approximation of theory and practice, and to deepen the meaning of opting for participation.

Cornwall^9^ suggests that participation is not merely a research technique, but a political process that addresses issues of inequity and social justice. It is understood that the essence of this approach is associated with a constructivist paradigm in which the specialist tries to stimulate the voice of people who do not participate or participate very little in the democratic process, playing a role of negotiation facilitator between the various points of view for the construction of an evaluation knowledge^16^.

Cousins and Whitmore^10^ describe three dimensions, later rearranged by Daigneault and Jacob^11^, for the analysis of the participation aspect in evaluations, which are: 1) the control of the evaluation process; 2) the diversity of participants; and 3) the extent of their involvement. It is thought that reflection on these aspects contributes to the identification of decisions and conditions that favor or limit participatory processes.

We highlight three reasons that justify the study and the use of the participatory methodology in health sector evaluations in Brazil:

1. The need for contextualized reflection and decentralization of decision-making for the regionalization process of the Unified Brazilian Health System (SUS)^19^, which should occur based on consistent and appropriate negotiations by local actors. Participatory evaluation is an opportunity to read and reflect on the real power of these actors to build a regional governance.
2. Public health evaluations are still very much identified with a narrow notion of accountability^7^ – a process of making the actors responsible for the evaluated policy – from which the quality of evaluation is judged in a way conditioned to the use of methods operated from of a positivist paradigm. It is necessary to deepen the understanding about the gain in quality in the processes evaluated, knowing that the evaluation will never be impartial and objective.
3. The understanding that care improvement comes from the active involvement of the citizen in the process of health production^21^. The evaluation is emphasized as an opportunity to develop and appropriate knowledge that has a meaning for the participants^22^, and that contributes to their influence during the evaluated programs or services^25^.

The objective is to critically analyze the “participation” criterion in a concrete experience of participatory evaluation, evidencing aspects that reinforced or limited this methodological option.

## METHODS

The meta-evaluation was developed based on workshops held with municipal managers and administrators from eight municipalities^a^ (out of a total of 18) from one of the selected Interagency Commission of the chosen health region (CIR), who voluntarily accepted the invitation sent from the research team to all participants.

Their task was to develop the evaluation of a specific aspect of their territory’s care line, in which the chosen theme was the flow of elective surgeries (cholecystectomy). The primary data collection for the meta-evaluation took place in the workshops, with an audio recording of the interactions among the participants, and in semi-structured interviews conducted by a professional outside the process after the activities were finalized.

The primary evaluation, carried out by the municipal representatives, was developed according to a participatory methodology, defined here by the promotion of a partnership relationship between participants and researchers for the development of an evaluative knowledge^8^.

Qualitative and quantitative methods were used, seeking to meet the information needs of the participants about the evaluated object.

The data for the quantitative approach were obtained from the information systems of the DATASUS, part of the Regulation Center for the Supply of Health Services (CROSS), and the municipal regulatory services; and the data analysis occurred in the workshops.

The qualitative approach was performed through five focus groups and six semi-structured interviews with users from five of the participating municipalities (totaling 29 people interviewed), and by an interview with the director of the Medical Specialist Ambulatory (AME). The inclusion criterion for users was the wait for the cholecystectomy surgery or its completion up to two years before in public services.

The qualitative data collected were organized by the facilitators according to the information they revealed about the trajectory of the users in the search for the necessary care and analyzed from a hermeneutic-dialectic methodology by the workshop participants. At the end of the process, a meeting was held in the technical chamber (CT) of the CIR and another in the CIR itself to give feedback on the evaluation findings.

All participants agreed to take part in the study and signed the terms of free and informed consent. The research was approved by the Ethics Committee of the Faculdade de Saúde Pública of the Universidade de São Paulo (Opinion 1.006.380).

## RESULTS AND DISCUSSION

The results are presented here according to the dimensions proposed by Cousins and Whitmore^10^, later reorganized by Daigneault and Jacob^11^, for the analysis of the “participation” aspect in evaluations, to qualify them in the developed initiative.

Complementarily, decisions and parts of discussions are summarized in the empirical material that signal aspects considered important to characterize a participatory process.

### Evaluation Process Control

This first dimension basically refers to the analysis of which groups or individuals have control over the technical decisions related to the evaluation.

The project under study was presented to the CIR and its participants opted to deepen the proposal in the CT, with subsequent resubmission at a general meeting. One measure, suggested and made effective by the CT group and which was one of the first examples of the shared decision, was to adapt the content of the proposal presented by the researchers to the target audience. “... *you need to tell managers what is the task, how often they have to come, what is the subject, what they will need to bring... I can already see us arriving at the CIR meeting with the proposal and nobody wanting to participate... It is important to say what they have to do*...” (Technical advisor 1).

The small group decided that one of its members, and not one of the researchers, would be responsible for initiating the resubmission of the proposal in the CIR in the format discussed. “*What I would like, as a manager, to contribute, is that the purpose of this study, which for the university is going to be a study, evaluation, for us will be a time to build a tool to evaluate our data... to learn to assess our needs*” (Municipal manager 1 during a discussion in the CIR). This was the start of a process of appropriation of the research that the managers went through during the entire study.

As of this new presentation, representatives from seven municipalities (including the central municipality of the region) were willing to participate directly in the process. With this group, seven workshops were held, distributed over 10 months of work, in which the evaluation process was built, focused on the meta-evaluation.

The decision about the evaluation theme was based on the discussion of a list of “annoyances” experienced by managers while performing their duties – especially in moments of decision making – sent by all members of the CIR to the CT. Demands and interests regarding possible aspects to be evaluated were contemplated, accompanied by a reflection on the governability of the actors for possible changes.

The need felt by the municipal managers for information regarding the flow between services of different federal levels was one of the demands that supported the choice of the evaluation theme: “*The individual is shared by different services, managers who are in different autonomous entities*...” (Municipal manager 5), “...*we do not know if he’s waiting here, if he’s waiting at AME*... *The user, besides being in the hands of two managers, one does not know what the other is doing…*” (Municipal manager 1), *“We end up needing information that we do not have access to and that we need to appropriate*...” (Municipal manager 3).

We observed that, in accordance with the participatory process, the order of stages usually followed for the development of an evaluation was not adopted in this process, although we believe that, in the end, all have been fulfilled.

In a first meeting of the group, for example, quantitative municipal data were analyzed – requested to the managers still in the CIR –, and we resumed the discussion about focus and evaluation questions initiated in the CT and discussed the insertion of users in the process.

Navigating the various interests while seeking to promote involvement and, at the same time, the quality of evaluation, was a continuous challenge, which is at the heart of a participatory approach^4^.

Keeping this process organization dynamic, all technical and operational decisions were made jointly by the facilitators and members of the management group.

### Participants Diversity

This analysis must consider the variation between the participation of only the primary users (usually funders and managers of the evaluated process) to the inclusion of all legitimate audiences.

The focus evaluation was developed by municipal managers and administrators. One of the factors that led the process to the maintenance of a homogeneous group of stakeholders was its initial orientation to a demand of these actors, which made the evaluation return to a question of managerial priority.

The theme chosen by them – the flow of elective surgeries (cholecystectomy) – ended up being closed to reflections and solutions that could only be sought by management. It was clear that the flow of care to the problem of biliary calculus had as its main bottleneck the ability to perform the referred surgery in referral hospitals (municipal and state), a matter whose referral was understood as the negotiation between levels of SUS management.

Respect for the choices in the participatory process (scope, theme, among others) limited, on the one hand, the diversity of participants in the evaluation. On the other hand, it favored the involvement of the managers “...*we were able to gather information, I went by myself to my Santa Casa (hospital), I talked about why we used this code more than another... our provider gave us a list of patients, how many were waiting for surgery... we had never had access to that information...*” (Municipal manager 1).

There was a question about whether there was a “tendency toward managerialism” in this evaluation, as described by Guba and Lincoln^16^. These authors point out that it may be more “comfortable” for those responsible for the evaluation to adopt normative methods and a preference for the inclusion of stakeholders with greater decision power, usually funders or managers.

However, it was considered that, although managers, these municipal actors were historically located in a place of less proactivity and representation in the CIR than their state partner, and that their strengthening as a homogeneous group would facilitate the understanding of the place occupied in the dynamics between management levels – the main objective when choosing the evaluation theme.

In addition, the participants did not represent a group external to the evaluated object. The performance of the departments under their responsibility, as organizers of care in the municipalities, was much discussed in the process.

To the extent that the decision was made to include the user’s perspective in the assessment, even bringing in the user as an informant in the data collection, operational difficulties had to be faced. Reports of difficulties finding and contacting these actors were frequent. There were also references to the lack of collaboration with other instances of the departments, which did not address our demand for the organization of focus groups. “*If I ask other coordinators or the people at the Family Health Program (FHP), they’ll say: <<I already have too much work, don’t give me any trouble...>>... The FHP coordinator acts as if it were a parallel directorate, he does not want to know about the health department. He is the vice-mayor’s brother and*...” (Technical advisor 1).

Nonetheless, participants’ insistence on listening to users led to the organization of focus groups and interviews in five of the seven participating municipalities. It was possible to state that the actor’s point of view was included in the study. As an example, the urgency in the shortening of the waiting time between the initial complaint and the surgery – given the painful physical and emotional experience lived by them, as well as the significant risk of worsening the situation – was greatly reinforced throughout the discussion between managers, as well as the need to transmit information and situate the user in terms of deadlines and call order to perform the surgery.

In the groups, as expected, the participants tended to focus on individual problems and showed, even when stimulated, difficulties in seeing issues that could be common to colleagues. We observed that the current SUS development process continues to bring users to a role of health technology consumers^12^, keeping them continuously away from the decision process. There is no identification in the organization of services, of any level of complexity in the region under study, of initiatives to make them part of the negotiations.

It should be noted that this trend may have been further accentuated by the focus on an acute health problem. A chronic problem requires systematic presence in the treatment unit, which increases the chance of greater attachment and appropriation of the service.

If the evaluation theme had been an open question to the construction of different referrals (it was not, as already mentioned, due to the real importance of the surgery), it would have required an investment in a framework and process of grouping, so that these actors could willing participate in the various stages of a service evaluation. This investment of time and resources should be predicted in such processes.

In the workshops, it was suggested that members of the unit’s Management Council should also be invited to the focus groups to broaden their understanding of the problem in question and open the possibility of their inclusion in the evaluation as another group of stakeholders. Some managers opposed this action, saying that the Councils formed in their municipalities had a strong partisan political bias, and would not add value to the ongoing process. “*The neighborhood association there is very politicized, you cannot count on them. I also don’t have a board council, but it is harder to set one up in a small municipality*...” (Municipal manager 1).

Others had a more enjoyable experience and were willing to make the invitation. “*The councils are heterogeneous... we need one that has a comprehensive vision... They theoretically represent the community... some are there for their own benefit*” (Administrator 2).

Thus, in three of the groups formed, there was the participation of council members. Previously informed that the activity would seek to better understand the pathways of the treatment in focus in the region, two of them positioned themselves as listeners and a third brought as contribution a personal experience related to the health problem addressed. We did not perceive a listening or intervention that denounced their role.

Primary care workers collaborated to identify and locate users. Again, the emphasis on the need to perform surgeries in the shortest possible time dominated the informal dialogues established between them – workers – and the research team, and no hook was identified at that time for the invitation to integrate them into the evaluative process.

In participatory evaluations, it is essential to reflect on which stakeholders participate in the processes and to what extent this participation occurs. From the provision of information and participation in the analysis of collected data to the elaboration of recommendations and decision making, several possibilities present themselves and are validated only in the process^17^.

The comparison between the reality of an initiative and ideal models of participation, as a rule, does not contribute to its development. When we aim at understanding the context in which it develops, we can identify real alternatives of participation.

### Extension of Involvement

This dimension refers to the number of stages of the evaluation in which there is the participation of stakeholders (non-evaluators). The participation of the group in the delimitation of the focus and parameters of the evaluation has already been detailed. Also, the selection of data to be collected was based strictly on the interest of the participants and on the joint judgment on which data would add value to understand the focus of the work, for example: “*We will access users of municipalities that have the municipal service, but who wait for the AME because they chose laparoscopy* (which is only offered at the State Hospital, unlike open surgery). *The importance of this is whether they are aware of the risk or not*...” (Municipal manager 3).

The exploitation of the information and regulation systems had the participation of all those involved. Access was done individually and collectively with the in-person support of a consultant in the workshops. “*For this reasoning, you must account for the urgency expressed by the city, the urgency expressed by the State, the urgency expressed by everyone... If the elective was offered in greater numbers, there would be less urgency.*..” (Municipal manager 1).

In the qualitative approach, users and the director of the AME were heard in focus groups and individual interviews organized and scheduled by the municipal teams, and performed by one of the facilitators to perform them in a timely manner. Listening to the interviewees was the only stage in which the managers or administrators did not participate.

The process of analysis and reflection on the collected empirical material occurred in the workshop immediately after the collection, to plan the next step. “*From the speeches* (focus groups)... *you see that they say that primary care is not so much the problem*...” (Administrator 2).

There were discussions and opinions of the process returned to the CIR group and to the work group composed of the CIR’s CT members, representatives of the state team of the territory and the social organization that manages them, and private providers. We considered that the main difference between the data construction appropriated by the participants is the possibility that they may use the process and evaluation findings in the various discussion and management forums when they deem it possible and necessary.

## CONCLUSIONS

The analysis of the participation dimensions makes clear the important presence and performance of stakeholders who are not evaluation specialists in the planning and development of this evaluation. This type of approach facilitates the identification of spaces and strategies focused on real problems of the stakeholders’ experience, allowing them to appropriate and take responsibility for the process to use this new knowledge in their daily life.

We observed that efforts were made to follow the movement of the group initially formed from the CIR, and not to shape the process according to the previously elaborated design. Thus, coherence was maintained in relation to the choice announced by a constructivist methodology and a feeling of belonging was generated in the participants.

Listening first to a determined group of stakeholders, working to strengthen their identity, brought advantages while engaging stakeholders and sustaining the project, and resulted in developing a continuous negotiation process. This process, in turn, allowed us to contemplate perspectives of analysis and action by people occupying distinct positions in the context of the health system.

The complexity of a participatory approach lies largely in its openness to encompass divergent interests, not easily circumvented in a process of limited duration. Therefore, it is important that the dynamics of the process can develop not only a consensus but also different views so that the group can feel contemplated in the chosen path.

When the evaluation process is driven by external resources, or even by internal resources, it is important that the work dynamics be based on flexibility and constant critical reflection with the effective participation of those responsible for the evaluation process and all members of the group. Thus, the process will provide real opportunities for participation and avoid the immobilization by waiting for ideal conditions to effectively mobilize resources that enable decisions resulting from the evaluation.

Decisions taken from reading the interface in this type of process have limits and scope that, coupled with contextual variables and legitimate interests of the participants, have to be accepted and problematized, and not just justified.

The work carried out allows us to infer that the participatory approach in a political process, such as the planning and evaluation of services, programs, and health systems, has a wide application in the sociocultural context of SUS implementation in the Brazil.

## Footnotes

a One of the managers who had been willing to participate was exonerated shortly before the start of the process, and thus the work was carried out with seven municipalities.
